# Subthreshold Psychiatric Psychopathology in Functional Gastrointestinal Disorders: Can It Be the Bridge between Gastroenterology and Psychiatry?

**DOI:** 10.1155/2017/1953435

**Published:** 2017-10-30

**Authors:** Cristina Stasi, Cristiana Nisita, Sonia Cortopassi, Giorgio Corretti, Dario Gambaccini, Nicola De Bortoli, Bernardo Fani, Natalia Simonetti, Angelo Ricchiuti, Liliana Dell'Osso, Santino Marchi, Massimo Bellini

**Affiliations:** ^1^Internal Medicine and Liver Unit, Department of Experimental and Clinical Medicine, Careggi University Hospital, Florence, Italy; ^2^Psychiatry Unit, Department of Clinical and Experimental Medicine, University of Pisa, Pisa, Italy; ^3^Functional Mental Health Unit of Adults-Northwest Tuscany Local Health Unit of Pisa, Pisa, Italy; ^4^Gastrointestinal Unit, Department of Clinical and Experimental Medicine, University of Pisa, Pisa, Italy

## Abstract

**Background and Aims:**

Functional gastrointestinal disorders (FGDs) are multifactorial disorders of the gut-brain interaction. This study investigated the prevalence of Axis I and spectrum disorders in patients with FGD and established the link between FGDs and psychopathological dimensions.

**Methods:**

A total of 135 consecutive patients with FGD were enrolled. The symptoms' severity was evaluated using questionnaires, while the psychiatric evaluation by clinical interviews established the presence/absence of mental (Diagnostic and Statistical Manual—4th edition, Axis I Diagnosis) or spectrum disorders.

**Results:**

Of the 135 patients, 42 (32.3%) had functional dyspepsia, 52 (40.0%) had irritable bowel syndrome, 21 (16.2%) had functional bloating, and 20 (15.4%) had functional constipation. At least one psychiatric disorder was present in 46.9% of the patients, while a suprathreshold panic spectrum was present in 26.2%. Functional constipation was associated with depressive disorders (*p* < 0.05), while functional dyspepsia was related to the current major depressive episode (*p* < 0.05). Obsessive-compulsive spectrum was correlated with the presence of functional constipation and irritable bowel syndrome (*p* < 0.05).

**Conclusion:**

The high prevalence of subthreshold psychiatric symptomatology in patients with FGD, which is likely to influence the expression of gastrointestinal symptoms, suggested the usefulness of psychological evaluation in patients with FGDs.

## 1. Introduction

Functional gastrointestinal disorders (FGDs), better defined as disorders of gut-brain interaction, are a combination of various chronic or recurrent gastrointestinal symptoms without a structural basis to explain their clinical features [1]. These include different disorders and involve the entire digestive system. The symptoms of these disorders result from a complex interplay among different and synergic factors such as alterations of gut microbiota modulation and mucosal immunity, visceral hypersensitivity, and central nervous system dysregulation of the modulation of gut signalling and motor function [1–4]. Given their relevance, these factors should be encompassed in the relatively new field of neurogastroenterology [5].

It is worth noting that these factors are the most frequently reported conditions by the gastroenterologists and constitute an important portion of the general practitioner's work; they cause significant absenteeism from work, decrease the health-related quality of life, and increase the medical costs [6, 7].

The cross-cultural aspects and psychological factors in FGD are well recognized [8]. The psychological and environmental factors together with the psychological distress affect both the clinical expression and symptom severity and thus play a cocausative role in the onset and course of FGDs in susceptible persons [2, 3, 9–13].

Anxiety, depression, and somatoform disorders are often reported in patients with FGD, especially in those reporting to gastroenterology clinics and/or referral centres. However, this contrasts studies reporting patients with organic gastrointestinal problems who exhibit a lower prevalence of psychiatric disorders [14, 15].

Patients tend to consider their psychological problems as a consequence of the same cause that produces FGDs. However, in almost half of the patients with a psychiatric disorder, the psychopathological symptoms start before the gastrointestinal signs, while they begin at the same time in large portion of the remaining patients [14, 16].

Although many studies demonstrated a strong association between FGDs, psychological disturbances, and social stress [9], relatively few studies directly reported a relationship between FGDs and a well-defined psychiatric diagnosis.

Recently, Sahoo and Padhy [8] reviewed the cross-cultural aspects and psychological factors in patients with IBS. They essentially indicated that carefully collecting the medical history and undertaking a holistic approach were relevant for the management of patients with IBS. Moreover, they outlined that a multidimensional approach, including both a psychiatric and gastroenterological assessment, is an essential part of an effective management of FGDs. In addition, on the basis of other multidisciplinary care models, a sharp decrease in health care resources should be expected because it could increase the efficacy and efficiency of the disease management, reaching a more rapid diagnosis, accelerating treatment planning, avoiding unnecessary duplication of tests, decreasing the patients' anxiety, and shortening the waiting times for receiving a coordinated plan of care [17].

Previously, the lack of reliable diagnostic criteria for classifying FGDs and mental disorders represented the most important methodological bias. In the last 20 years, the introduction of the DSM system and the Rome criteria strongly contributed to clarify better this intriguing matter.

Studies [14, 18–20] assessing the comorbidity of psychiatric symptoms and FGDs applied a traditional categorical diagnosis according to the different systems of classification of mental disorders (International Classification of Diseases [ICD-9], ICD-10, DSM-III-revised, and DSM-IV). Although these studies significantly contributed to the specification of this relationship, the categorical view of mental illness risks did not consider a large part of psychopathological signs and symptoms under the diagnostic threshold that may influence clinical management and prognosis of FGDs, such as other medical and psychiatric disorders.

The concept of spectrum disorders [21] has been developed in order to identify the whole amount of psychopathological signs and symptoms characterizing a mental disorder more efficiently. In addition to typical DSM core symptoms, the spectrum comprises isolated or atypical symptoms, often of low severity, as well as trait-behavioural features, which tend to persist, waxing and waning, through the lifespan. This use of prodromal, subthreshold, typical, atypical, residual, and trait-like symptoms allows a dimensional evaluation of anxiety and mood phenomena and a better comprehension of the psychopathological continuum [22–24].

Therefore, several assessment instruments, including structural interviews and self-report questionnaires, were developed and tested in recent years [25].

Based on these observations, the aims of this study were to investigate the prevalence of Axis I and spectrum disorders in a population of patients with FGD and to establish the possible links between some FGDs and some psychopathological dimensions.

## 2. Patients and Methods

From 1 February 2013 to 31 July 2014, 135 consecutive patients with FGD (34 men [25.2%], 101 women [74.8%]; mean age 43.22 ± 15.05 years; age range 18–70 years), referred to the gastroenterology outpatient services of the Gastrointestinal Unit of the University of Pisa, Italy, were enrolled in this study.

The inclusion criteria were as follows: (1) patients referred for functional gastrointestinal disorders at the Gastroenterology Unit and (2) age range 18–70 years.

The following exclusion criteria were considered: (1) severe organic diseases; (2) significant changes in serum chemistry; (3) history of abdominal surgery (except appendectomy); (4) lactose intolerance (demonstrated by lactose breath test); (5) pregnancy; and (6) medication taken within 15 d prior to the study, which alters the autonomic response (e.g., anticholinergic drugs or beta blockers), psychotropic activity (e.g., tricyclic antidepressants, serotonin [5-HT] reuptake inhibitors, or benzodiazepines), and/or that potentially interfere with the gastrointestinal motility.

The study protocol was approved by the Ethical Committee of Pisa and was carried out in accordance with the tenets of the Helsinki Declaration (6th Revision, Seoul, 2008). Furthermore, written informed consent was obtained from each participant.

### 2.1. Gastroenterological Evaluation

The patients were grouped in accordance with their FGD: 42 (32.3%) were diagnosed with functional dyspepsia (FD), 52 (40.0%) with irritable bowel syndrome (IBS), 21 (16.2%) with functional bloating (FB), and 20 (15.4%) with functional constipation (FC).

The diagnosis of the different FGDs was performed in accordance with the Rome III criteria [26, 27].

The severity of the symptoms was evaluated using specific questionnaires including the IBS Symptom Severity Score (IBS-SSS) to evaluate abdominal symptom severity [28] and the Dyspepsia SSS [29]. Additionally, a “homemade” bowel habits questionnaire using a scale ranging from 0 (no symptoms) to 4 (symptoms present during ≥75% of bowel movements or days) [30] evaluated the frequency of (1) painful defecation, (2) manual manoeuvres facilitating defecation, (3) hard faeces, (4) watery faeces, (5) “fragmented” defecation, (6) sensation of anorectal blockage, (7) urge to defecate, (8) incontinence for gas and/or faeces, (9) abdominal pain, and (10) abdominal bloating.

The degree of interference of the gastrointestinal symptoms with the global well-being was expressed by a visual analogue scale (0–100 mm), with 0 indicating *absent* and 100 indicating *unbearable*.

### 2.2. Psychiatric Evaluation

The psychiatric evaluations were based on structured clinical interviews aimed to establish essentially the presence or absence of (1) mental disorders (DSM-IV Axis I diagnoses) and (2) spectrum disorder.

### 2.3. Instruments

#### 2.3.1. Structured Clinical Interview for DSM-IV Patient Edition (SCID IV)

The Structured Clinical Interview for DSM-IV Patient Edition (SCID IV) [31] was administered to diagnose Axis I disorders using the DSM-IV categorical criteria. SCID-IV is a hetero-evaluation instrument used to assess psychiatric Axis I disorder into different nosographic issues. The interview consists of a first part aimed to reveal the demographic data, such as work and educational level, as well as medical and pharmacological history. The second part consists of different sessions: mood disorders, episodes (session A), psychotic symptoms associated to mood disorders (session B), psychotic disorders (session C), mood disorders, course (session D), substance abuse disorders (session E), anxiety disorders (session F), somatoform disorders (session G), eating disorders (session H), and adjustment disorders (session I).

#### 2.3.2. Structured Clinical Interview for Panic-Agoraphobic Spectrum—Self-Report Version (SCI PAS-sr)

SCI-PAS [32] is a clinical interview aimed to evaluate the presence or absence of panic and agoraphobic spectrum symptomatology. It consists of 114 items grouped into 8 domains: (1) separation sensitivity; (2) panic-like symptoms (typical and atypical); (3) stress sensitivity; (4) substance and medication sensitivity; (5) anxious expectation; (6) agoraphobia; (7) illness-related phobia; and (8) reassurance orientation. Every item was coded as “true” (1) or “false” (0), and the diagnostic threshold was 35.

#### 2.3.3. Structured Clinical Interview for Mood Spectrum—Self-Report Version (SCI MOODS-sr)

SCI-MOOD [33] consists of 140 dichotomous items aimed to evaluate the presence/absence of mood spectrum signs and symptoms and grouped into seven domains: (1) mood depressed; (2) mood manic; (3) energy depressed; (4) energy manic; (5) cognition depressed; (6) cognition manic; and (7) rhythmicity and vegetative functions. The diagnostic threshold was 61.

#### 2.3.4. Structured Clinical Interview for Anorexic-Bulimic Spectrum—Self-Report Version (SCI ABS-sr)

SCI ABS-sr [34] is a clinical interview aimed to evaluate the presence or absence of anorexic-bulimic spectrum symptomatology. It consists of 134 items grouped into nine domains: (1) attitudes and beliefs; (2) history of weight loss; (3) self-esteem and satisfaction; (4) phobias; (5) avoidance and compulsive behaviours; (6) weight maintenance; (7) eating disorders; (8) associated features and consequences; and (9) impairment and insight. The diagnostic threshold was 45 points where the answer coded by *yes* was equal to 1.

#### 2.3.5. Structured Clinical Interview for Obsessive-Compulsive Spectrum—Self-Report Version (SCI OBS-sr)

SCI OBS [35] is a clinical interview aimed to evaluate the presence/absence of obsessive-compulsive spectrum symptoms. The SCI-OBS consists of 196 items grouped into seven domains: (1) childhood/adolescence experiences; (2) doubt; (3) hypercontrol; (4) attitudes toward time; (5) perfectionism; (6) repetition and automation; and (7) specific themes. Every item was coded as present or absent, and the diagnostic threshold was 59.

#### 2.3.6. Structured Clinical Interview for Social Anxiety Spectrum—Self-Report Version (SCI SHY-sr)

SCI SHY [35] is a clinical interview aimed to evaluate the presence/absence of social anxiety spectrum symptoms and consists of 164 items grouped into four domains: (1) social phobic traits during childhood and adolescence; (2) interpersonal sensitivity; (3) behavioural inhibition and somatic symptoms; and (4) specific anxiety and phobic features. The appendix explores the use of psychoactive substances, which is a frequent complication of the social anxiety disorder. The diagnostic threshold was 59.

#### 2.3.7. Statistical Analysis

All data were analysed using SPSS (SPSS Inc., Chicago, IL, USA) and Stata 12 software (Stata Statistical Software, College Station, TX, USA). All variables are expressed as the mean ± standard deviation. The numerical comparison of the continuous data was performed using the *t*-test for paired samples. Statistical significance was set at a value of *p* < 0.05. The Chi-square test was used to evaluate the categorical variables. Linear regression analysis between two variables was performed using Pearson correlation.

We have performed the multinomial logistic regression analysis to evaluate the associations between the global well-being and spectrum disorders/Axis I diagnosis, the well-being in IBS patients and spectrum disorders/Axis I diagnosis, the well-being in FB patients and spectrum disorders/Axis I diagnosis, the well-being in FD patients and spectrum disorders/Axis I diagnosis, and the well-being in FC patients and spectrum disorders/Axis I diagnosis. In this analysis, the score ≥ 40 was considered interfering with well-being.

## 3. Results

Table 1 summarizes the functional gastrointestinal diagnosis groups of patients with FDGs.

At least one diagnosis of the psychiatric Axis I disorder was present in 46.9% of the patients. In particular, the generalised anxiety disorder was diagnosed in 20.0% of the patients, while panic disorders, including panic disorder, panic without agoraphobia, and agoraphobia without panic attack, were diagnosed in 27.7% of the patients. Mood disorders were the most frequent and were diagnosed in 13.1% of the patients (Table 2).

Regarding spectrum symptomatology, a suprathreshold panic spectrum was present in 26.2% of the patients. Despite the low prevalence of Axis I obsessive-compulsive disorder (0.8%) and social phobia (1.5%), a high percentage of the patients exhibited a positive obsessive (19.2%) and social phobic (23.8%) spectrum. Of the entire population, 39.2% had at least one spectrum symptomatology of anxiety, while 43.1% had at least one spectrum symptomatology of affective disorders.

A low prevalence (3.9%) of axis eating disorders (anorexia, bulimia, and binge eating disorder) and a higher (15.4%) suprathreshold spectrum symptomatology were also observed.

Almost 13.1% of the patients had an Axis I mood disorder, while 20.8% had a positive mood spectrum (Table 2).

Table 3 summarizes the prevalence of psychiatric disorders and spectrum symptomatology in the different FGDs. Significant associations were observed between FGDs (in particular FC) and Axis I diagnosis depressive disorders (dysthymia and recurrent depression; *p* < 0.05), while current major depressive episode was significantly related to FD (*p* < 0.05).

When we considered the relationship between FGDs and spectrum disorders, obsessive-compulsive spectrum was significantly related to the presence of FC and IBS (*p* < 0.05).

Considering the severity of the gastrointestinal symptoms and the degree of interference with the global well-being of the patients, a social phobic spectrum showed a significant correlation with IBS (*p* < 0.05). Indeed, in the IBS group, the comorbidity with social phobia was related to a lower level of well-being, particularly with symptoms such as defecation urgency (*p* < 0.01) and abdominal pain relieved by evacuation (*p* < 0.05). However, the presence of spectrum disorders was positively related to the degree of interference of the gastrointestinal symptoms with the subjective global well-being. Thus, patients without an Axis I disorder or a suprathreshold spectrum had a significantly lower level of interference with the global well-being (*p* < 0.05).

Moreover, the presence of obsessive, social phobic, and eating positive spectrums significantly decreased the global well-being score. Obsessive and eating spectrums were directly related to the well-being impairment of the IBS group, while the social phobic spectrum was related to the impairment of the well-being of the FC and FB groups. A positive mood spectrum was inversely related to the well-being of the patients with IBS (Tables 4 and 5).

When we performed the multinomial logistic regression analysis, we found a significant association between the global well-being and spectrum disorders (*p* = 0.01), but we found no significant association between global well-being and Axis I diagnosis (*p* = 0.26). When we considered the different FGD groups, we found a significant association between well-being in FC patients and spectrum disorders (*p* = 0.03).

## 4. Discussion

The relationship between FGDs and psychiatric disorders has been widely studied, but the results have been often conflicting, mainly because of the complexity of the question and the methodological bias in many studies.

Our study fully reflected the disputes concerning the intriguing question “irritable brain or irritable bowel.” The results confirmed a high psychiatric comorbidity in FGDs, particularly mood and anxiety disorders, in the sense of full diagnosis (one-third of the sample met the criteria for the Axis I mental disorder).

However, independently of the full-blown diagnosis, a particular value of this study was the organisation for analysis, using appropriate standardized instruments, of a psychopathological dimension, often labelled as “vulnerability psychological features” and observed initially by gastroenterologists.

For the first time, this study analysed the subthreshold psychiatric symptomatology in patients with different FGDs. It also confirmed the previous observations about the high prevalence of Axis I psychiatric disorders among patients with FGD [3, 4, 9, 17, 30, 36], particularly anxiety and mood disorders. Subthreshold psychiatric symptomatology is formed by a variety of symptoms that do not conform to a formal diagnosis of mental disorder according to standard psychiatric classification, but it impacts significantly in quality of life.

However, the presence of a psychiatric Axis I disorder seemed to explain partly the complex relationship between the brain and the gut, particularly the clinical expressivity of the functional gastrointestinal disorders. In previous studies [3, 4, 36], investigating psychological disorders, we found a high prevalence of psychological disorders, but we have also found that many patients had elevated scores for psychopathology, however without reaching a significant score for diagnosis. This led us to investigate spectrum disorders in patients with FGDs. The comorbidity between FGDs and psychiatric disorders is not simply restricted to Axis I disorders but it also widely extends to spectrum psychopathology, such as subclinical, prodromal, or residual symptoms. Spectra instruments permit the collection of underlying soft psychopathological symptoms generally defined as neurotic traits or stress-related symptoms. Spectrum diagnoses showed a strong coherence with DSM-IV diagnoses and provided additional clinical information. It is likely that this assessment can be used in patients with nonpsychiatric disorders to provide a more comprehensive picture of symptoms and behavioural traits. Additionally, previous studies indicated that the presence of spectrum symptomatology has a clinical relevant significance as a predictor of outcome in major depressive and bipolar disorders [37].

In this study, the use of the spectrum instruments has enabled pointing out to the presence of a subthreshold psychopathology in 44.6% of the study sample, compared with an almost overlapping percentage of diagnosis of Axis I (46.9%). It is worth noting that a spectrum diagnosis was also present in 27.5% of patients with no major psychiatric disorder; therefore, these spectrum instruments are also very useful in patients with nonpsychiatric disorders not affected by Axis I disorders, for whom a traditional psychiatric approach is not feasible.

The distribution of spectrum diagnoses in our sample (PAS 26.2%, MOOD 20.8%, OBS 19.2%, SHY 23.8%, and ABS 15.4%) demonstrated an affective continuum that characterized the dimensional psychopathological profile of this cohort with clinical nonpsychiatric disorders. Our data further indicated a close relationship between FGDs and anxiety spectrum symptomatology, such as that underlined by the high prevalence of panic-agoraphobic, social phobic, and obsessive-compulsive spectrums. In consideration of this, the unspecific terms of “neuroticism” and “stress reaction” often used to label patients with FGDs in the past may be replaced by the term “anxious spectra.”

However, the low-grade inflammation associated with the release of interleukin- (IL-) 1, IL-6, and tumour necrosis factor- (TNF-) *α* may activate the cerebral circuits via afferent fibres [2]. Indeed, recent findings demonstrated that the gut microbiota plays a pivotal role in stress-related psychiatric disorders [38, 39] and that the sensitivity and gastrointestinal motility are both regulated by the central and peripheral nervous systems via the complex interplay between the brain and gut [3, 4]. 5-HT plays a key role in the regulation of visceral pain and in the secretion and initiation of the peristaltic reflex. However, altered levels of 5-HT are also detected in many different psychiatric disorders such as anxiety, depression, obsessive-compulsive disorders, and phobia [13, 17]. The 5-HT released in the gut from the enterochromaffin cells regulates the sensory, motor, and secretory functions of the digestive system through interactions with intrinsic and extrinsic nervous pathways. Intrinsic innervation to the gut is supplied by neurons of the ENS, including the myenteric and submucosal plexus. Extrinsic innervation is provided by the autonomic nervous system (both sympathetic and parasympathetic) and is arranged to function in a bidirectional manner [11, 40, 41]. Moreover, the 5-HT concentrations are regulated by its reuptake, which is operated by the 5-HT transporter (SERT) expressed both in neurons and intestinal epithelium [40–43].

Our data demonstrated that the presence of spectrum diagnoses influences the clinical picture of FGDs, particularly well-being, quality of life, and severity of the FGD symptoms.

Spectrum diagnoses tend to be related to a low well-being referred by patients and to a higher severity of FGD symptoms. In fact, the presence of spectrum disorders, in particular in FC patients after multivariate analysis, was positively associated with the degree of interference of the gastrointestinal symptoms with the subjective global well-being. This could indicate that psychic dimension may change the perception of the symptoms.

This is possibly owing to the psychological disorders that in turn lead to a chronic state of amplification of symptoms, which originates either at the level of the CNS (hypervigilance on physical perception) or at the visceral level (hypersensitivity and hypermotility) [2].

Obsession, which is revealed by a positive obsessive-compulsive spectrum, was correlated with FGDs such as FC and IBS.

Interesting conclusions could be inferred from the second part of the analysis regarding the spectrum psychopathology, severity, and global well-being both in the whole population and in the FGD groups. Higher gastroenterological symptomatic scores with a social phobic spectrum were detected in patients with IBS compared to the other FGD groups. Social phobic psychopathological nucleus is based on the overestimation of judgement and criticism from others; it may be reflected by an increase of frequency and severity of anxiety-mediated IBS symptoms. The correlation with defecation urgency and pain relief after evacuation seems to confirm this view. Indeed, the fear of decontrol in the sphincter functions, which significantly increases the defecation urgency, and the significant decrease in anxiety levels after evacuation could be perceived by the patients as a reduction in the pain and urgency.

The obsessive and social-phobic dimensions were shown to be associated with “poor well-being”; thus, with a worse quality of life, they were assessed as subjective satisfaction and interference. However, PAS, MOOD, and ABS spectrum diagnoses as well as Axis I of panic attack disorder (DAP) generalised anxiety disorder and major depression; although they were easier to collect in nonpsychiatric clinical practice, they do not show equal predictive powers.

In particular, the well-being of patients with FGDs was significantly impaired when the gastrointestinal disease was associated with a positive obsessive and social phobic spectrum.

Obsessive patients seemed to be highly limited in their functioning by this reactivity and instability in evacuation, thereby triggering an inhibitory behaviour and a reduction in the quality of life. The association between low well-being in patients with FB and social phobic spectrum underlines the inhibited behaviour due to the fear of emitting intestinal sounds in public.

Especially spectrum diagnosis interfered negatively with the global well-being of the patients affected by FGDs, regardless of the specific FGD type and the severity of the gastrointestinal symptoms.

Subjective satisfaction, quality of life, and global well-being are variables that may increase the level of sufferance referred to a symptom, and they could also inspire the patients to refer to a physician or a gastroenterologist.

## 5. Conclusions

In conclusion, the high presence of subthreshold psychiatric symptomatology in patients with FGDs suggested the usefulness of psychological evaluations in these patients, in whom probably a brief self-evaluation scale could be used in the clinical gastroenterological practice, targeted to reveal subclinical psychopathological features that are likely to influence significantly the expression of the gastrointestinal symptoms. The second step could be represented by the evaluation of Axis I psychiatric disorders and the global level of well-being. Further studies are needed to clarify further the pathophysiology of psychological disorders often associated with FGDs.

## Supplementary Material

Symptom Severity Score Questionnaire.

## Figures and Tables

**Table 1:** Distribution (*N*, %) of different FGDs studied.

Functional gastrointestinal disorders	(*N* : 135)	%
Functional constipation	20	15.4
Irritable bowel syndrome	52	40.0
Functional dyspepsia	42	32.3
Functional bloating	21	16.2

**Table 2:** Axis I and mood spectrum disorders in patients with functional gastrointestinal disorders.

Axis I diagnosis	(*N*)	%	Spectrum	Mean + SD	Overthreshold
Anxiety disorders					
Panic	24	18.5	SCI-PAS	25.99 ± 17.54	26.2 (34)
Panic with agoraphobia	10	7.7			
Agoraphobia without panic attack	2	1.5			
Single phobia	4	3.1			
Anxiety NAS	4	3.1			
GAD	26	20.0			
Social phobia	2	1.5	SCI-SHY	39.47 ± 31.07	23.8 (31)
OCD	1	0.8	SCI-OBS	39.64 ± 24.69	19.2 (25)
Somatoform disorders	2	1.5			
Mood disorders					
Past major depressive episode	13	10.0	SCI-MOODS	38.25 ± 24.45	20.8 (27)
Current major depressive episode	2	1.5			
Recurrent depression	1	0.8	Dep	24.78 ± 17.24	
Dysthymia	1	0.8	Man	13.48 ± 9.55	
Somatization disorders	1	0.8			
Eating disorders					
Anorexia	1	0.8	SCI-ABS	20.53 ± 19.66	15.4 (20)
Bulimia	4	3.1			
BED	0	0.0			

SCI-PAS: Structured Clinical Interview for Panic-Agoraphobic Spectrum—self-report version; SCI-SHY: Structured Clinical Interview for Social Anxiety Spectrum; SCI-OBS: Structured Clinical Interview for Obsessive-Compulsive Spectrum; SCI-MOODS: Structured Clinical Interview for Mood Spectrum; SCI-ABS: Structured Clinical Interview for Anorexic-Bulimic Spectrum.

**Table 3:** The prevalence of psychiatric disorders and spectrum symptomatology in the different FGDs.

	FC % (*N* = 20)	IBS % (*N* = 52)	FD % (*N* = 42)	FB % (*N* = 21)
*At least 1 Axis I disorder*	60.0 (12)	36.5 (19)	52.4 (22)	47.6 (10)
Panic	25.0 (5)	17.3 (9)	19.0 (8)	14.3 (3)
Panic with agoraphobia	15.0 (3)	3.8 (2)	9.5 (4)	4.8 (1)
Agoraphobia without panic	5.0 (1)	0.0 (0)	2.4 (1)	0.0 (0)
Single phobia	5.0 (1)	1.9 (1)	2.4 (1)	4.8 (1)
Anxiety NAS	5.0 (1)	1.9 (1)	0.0 (0)	9.5 (2)
GAD	20.0 (4)	17.3 (9)	26.2 (11)	14.3 (3)
Social phobia	5.0 (1)	1.9 (1)	0.0 (0)	0.0 (0)
OCD	0.0 (0)	0.0 (0)	2.4 (0)	0.0 (0)
Somatoform disorders	5.0 (1)	1.9 (1)	0.0 (0)	0.0 (0)
Past MDE	9.5 (2)	11.5 (6)	9.5 (4)	14.3 (3)
Current MDE	0.0 (0)	0.0 (0)	4.8 (2)	0.0 (0)
Recurrent depression	5.0 (1)	0.0 (0)	0.0 (0)	0.0 (0)
Dysthymia	5.0 (1)	0.0 (0)	0.0 (0)	0.0 (0)
Somatization disorders	0.0 (0)	0.0 (0)	2.4 (1)	0.0 (0)
Anorexia	0.0 (0)	0.0 (0)	2.4 (1)	0.0 (0)
Bulimia	5.0 (1)	1.9 (1)	2.4 (1)	4.8 (1)
BED	0.0 (0)	0.0 (0)	0.0 (0)	0.0 (0)
*Spectrum symptomatology*				
Overthreshold SCI-OBS	35.0 (7)	9.6 (5)	19.0 (8)	23.8 (5)
Overthreshold SCI-SHY	25.0 (5)	21.2 (11)	31.0 (13)	14.3 (3)
Overthreshold SCI-PAS	30.0 (6)	21.2 (11)	28.6 (12)	23.8 (5)
*At least 1 anxiety spectrum*	50.0 (10)	32.7 (17)	42.9 (18)	33.3 (7)
Overthreshold SCI-ABS	25.0 (5)	9.6 (5)	16.7 (7)	14.3 (3)
Overthreshold SCI-MOODS	35.0 (7)	13.5 (7)	28.8 (10)	14.3 (3)

NAS: anxiety in autistic adults; GAD: generalized anxiety disorder; OCD: obsessive-compulsive disorder; MDE: mood disorder event; BED: bipolar disorder event; SCI-PAS: Structured Clinical Interview for Panic-Agoraphobic Spectrum—self-report version; SCI-SHY: Structured Clinical Interview for Social Anxiety Spectrum; SCI-OBS: Structured Clinical Interview for Obsessive-Compulsive Spectrum; SCI-MOODS: Structured Clinical Interview for Mood Spectrum; SCI-ABS: Structured Clinical Interview for Anorexic-Bulimic Spectrum; FC: functional constipation; IBS: irritable bowel syndrome; FD: functional dyspepsia; FB: functional bloating.

**Table 4:** Differences in FDGs between nonpsychiatric patients and patients with the presence of Axis I and/or spectrum disorders.

	Healthy	Axis I and/or spectrum disorders	*p*
Global well-being	36.70	31.50	<0.05
IBS well-being	17.40	8.75	<0.01
FB well-being	5.70	4.19	ns
Dyspepsia well-being	10.90	12.06	ns
FC well-being	2.70	6.50	ns

**Table 5:** Difference between psychiatric disorders (DSM IV) versus spectrum diagnosis stratified for well-being.

	OBS	SHY	PAS	MOODS	ABS
Global well-being	26.80^∗∗^	27.58^∗∗^	32.79	29.07	27.25^∗^
IBS well-being	4.60^∗∗^	10.32	8.24	6.30^∗^	4.00^∗∗^
FB well-being	5.20	1.61^∗^	2.50	4.63	2.50
FD well-being	11.20	13.70	13.82	11.85	13.50
FC well-being	5.80	1.94^∗^	8.23	6.30	7.25

^∗^ *p* < 0.05; ^∗∗^*p* < 0.01.
