# Supplementary figures and images for: Subthreshold Psychiatric Psychopathology in Functional Gastrointestinal Disorders: Can It Be the Bridge between Gastroenterology and Psychiatry?
Source: Gastroenterol Res Pract. 2017 Oct 30;2017:1953435. doi: 10.1155/2017/1953435 (PMC5682902; doi:10.1155/2017/1953435)

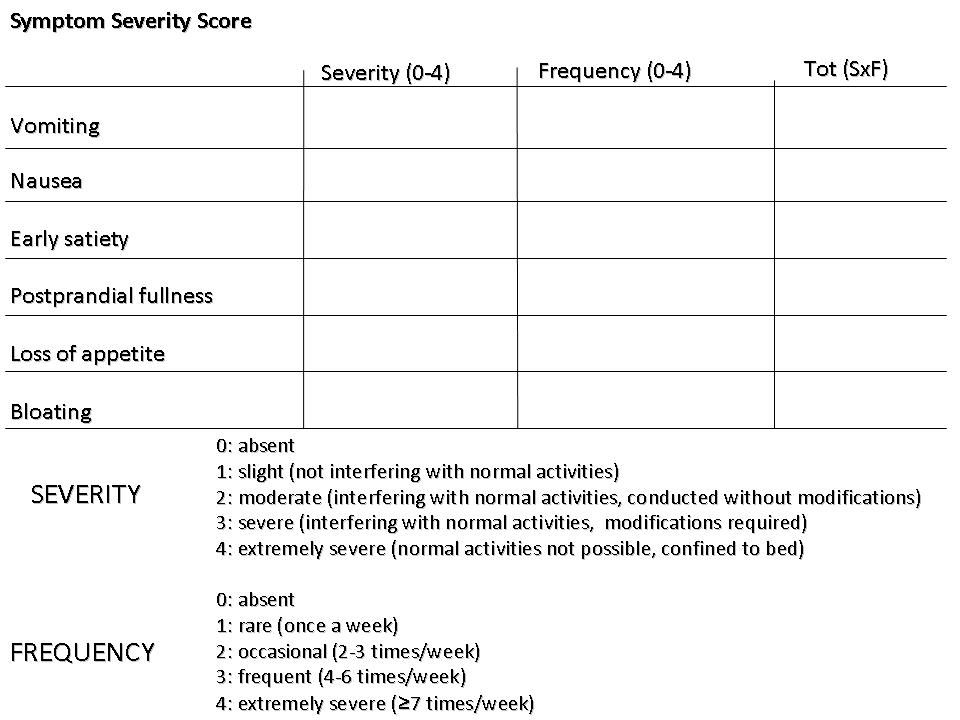

Supplement: Supplementary file 1 — Symptom Severity Score Questionnaire. [file 1953435.f1.jpg]
